# Relationship between thyroid hormones and bronchopulmonary dysplasia in extremely and very preterm infants

**DOI:** 10.3389/fphys.2026.1818723

**Published:** 2026-04-13

**Authors:** Yizhe Ma, Guihua Liu, Hu Li, Zhidan Bao, Xianhui Deng, Mingyan Tao, Luchun Wang

**Affiliations:** Department of Pediatrics, Jiangyin People’s Hospital of Nantong University, Jiangyin, China

**Keywords:** bronchopulmonary dysplasia, infant, neurodevelopment, preterm, thyroid hormone

## Abstract

**Aims:**

Thyroid hormones (THs) play a vital role in neonatal development. The present study aimed to evaluate the association between thyroid function and bronchopulmonary dysplasia (BPD) incidence as well as neurodevelopment in extremely and very preterm infants.

**Methods:**

This retrospective study involved 125 preterm infants born between 26 and 32 weeks of gestation from January 2020 to October 2025. Thyroid function tests were performed at 2 and 4 weeks after birth, and neurodevelopment was assessed using General Movements (GMs) at 36 weeks and 40 weeks of postmenstrual age (PMA).

**Results:**

Among the 125 infants, 40 infants (32%) developed BPD; of these 40 infants, 27 and 13 infants were classified as grade 1 and grade 2 BPD, respectively. Notably, the grade 2 BPD group had significantly lower TH levels compared to both grade 1 BPD and non-BPD groups (*P* < 0.05). Additionally, the non-BPD and grade 1 BPD groups showed a tendency to exhibit better neurological development at 36 and 40 weeks PMA compared to the grade 2 BPD group, although this difference was not significant.

**Conclusions:**

Serial thyroid function monitoring during the first month of life may be useful in identifying extremely and very preterm infants who are most at risk of developing severe BPD (grade 2). Future intervention studies are needed to determine whether thyroid replacement therapy in this particular high-risk group may reduce the severity of BPD and potentially improve neurodevelopmental outcomes.

## Introduction

Bronchopulmonary dysplasia (BPD) is a common complication of preterm birth, and it can lead to long-term respiratory disorders (Abiramalatha et al., 2022). Although advancements in neonatal care have improved the outcome for premature infants, BPD remains a persistent issue with an uncontrolled incidence rate (Jensen et al., 2019). BPD increases the risk of developing other major neonatal morbidities, and contributes to a higher in-hospital mortality rate and a greater need for respiratory support after discharge, which further leads to higher healthcare costs (Jensen et al., 2021). Several risk factors have been linked with the onset of BPD, such as preterm delivery, low birth weight, hereditary susceptibility, prolonged mechanical ventilation, hyperoxia exposure, and perinatal infection (Ryan et al., 2023). In the United States, more than half of preterm infants born at < 32 weeks of gestational age (GA) did not survive to 36 weeks of postmenstrual age (PMA) or developed BPD (Jensen et al., 2021).

The assessment of thyroid function in preterm neonates is critical because these infants are susceptible to thyroid dysfunction, and the immaturity of the hypothalamic-pituitary-thyroid axis in preterm infants often leads to conditions such as hypothyroxinemia, which is associated with delayed neurodevelopmental outcomes (Klosinska et al., 2022). Thyroid hormones (THs), particularly thyroxine (T4) and triiodothyronine (T3), are the key regulators of neonatal development (Vamesu et al., 2023). Transient hypothyroxinemia is considered a common epiphenomenon of non-thyroidal illnesses in premature infants (LaFranchi, 2021). However, it was reported as a risk factor for respiratory morbidities in moderately preterm neonates; moreover, emerging evidence suggests that neonatal hypothyroidism may be associated with an increased risk of BPD in premature infants (Du et al., 2017; Yoon et al., 2019). Although hypothyroxinemia and BPD have been linked in earlier research, our study provides a more detailed analysis by looking at a wide panel of thyroid parameters at 2 and 4 weeks postnatally, specifically concerning the 2019 Jensen BPD severity grades (Vamesu et al., 2023). In this retrospective study, total T3 (TT3), total T4 (TT4), free T3 (FT3), FT4, and TSH levels were analyzed to explore the relationship between thyroid function and BPD as well as neurodevelopmental outcomes in preterm infants born with gestational age (GA) < 32 weeks. Gaining insights into these relationships could improve clinical management strategies, ultimately enhancing the health outcomes for preterm infants with BPD.

## Methods

### Study population

**Figure 1:**
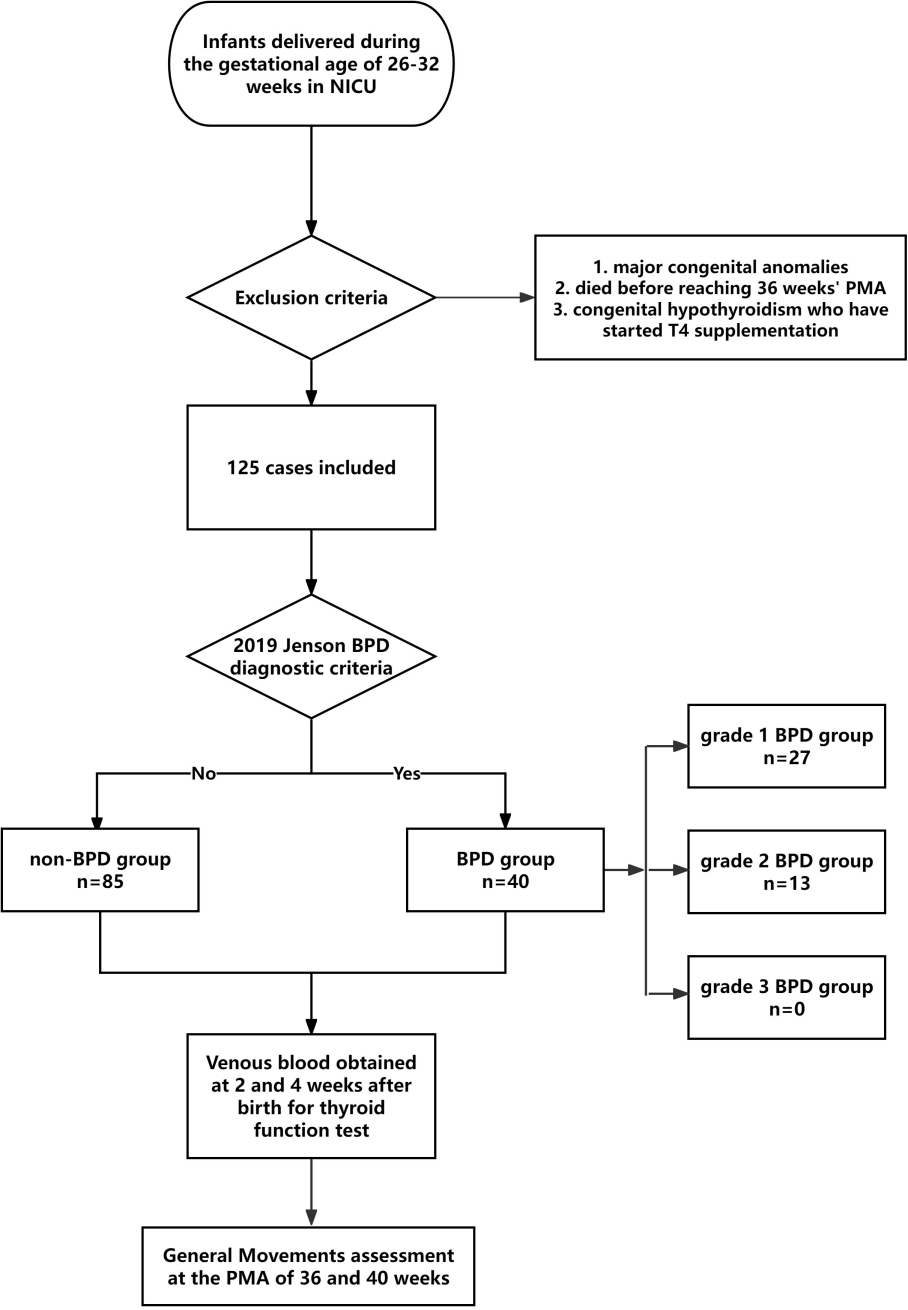
The flowchart of the study. PMA, Postmenstrual age; BPD, Bronchopulmonary dysplasia; NICU, neonatal intensive care unit.

This study retrospectively evaluated extremely and very preterm infants delivered between 26 and 32 weeks of gestation in the neonatal intensive care unit (NICU) of Jiangyin People’s Hospital of Nantong University from January 2020 to October 2025. Infants with major congenital anomalies or died before reaching 36 weeks’ PMA, or those diagnosed with congenital hypothyroidism who have started T4 supplementation were excluded from the study. We collected clinical data from obstetric and neonatal records, and participants received standard clinical care in hospital. Finally, 125 cases were analyzed. Venous blood was obtained at 2 weeks and 4 weeks post-birth for thyroid function test until the participants remained in the NICU. BPD was defined according to the 2019 Jenson BPD diagnostic criteria (Jensen et al., 2019). Accordingly, infants not requiring any supplemental respiratory support at 36 weeks of PMA were included in the non-BPD group; furthermore, infants treated with nasal cannula, those who received noninvasive positive airway pressure, and those treated with invasive mechanical ventilation were classified as grade 1 BPD, grade 2 BPD, and grade 3 BPD groups, respectively (Jensen et al., 2019). The flowchart of the study was shown in **Figure 1**.

Based on the reference ranges for term infants, overt primary hypothyroidism was defined by a low FT4 concentration and a concurrently elevated TSH level; however, we recognize that there are no established reference intervals for preterm infants (Rose et al., 2023). Sepsis was defined as the presence of positive blood cultures in a child with clinical signs and symptoms consistent with systemic infection, such as temperature instability, respiratory distress, or haemodynamic compromise (Celik et al., 2022). The evaluation of echocardiography was conducted by a pediatric cardiologist, and hemodynamically significant patent ductus arteriosus (HSPDA) was diagnosed by a combination of clinical observations and echocardiographic measurements (Mitra et al., 2023). Intraventricular hemorrhage was recognized as the Papile stage III or higher (Bell et al., 2022). Necrotizing enterocolitis was identified as the BELL stage 2 or higher (Bell et al., 2022). Trained researchers collected detailed maternal and infant information to analyze the clinical and laboratory characteristics of infants. The study was approved by the Institutional Ethics Committee of Jiangyin People’s Hospital (Approval No. 2024-L097).

### Thyroid function test

The NICU followed a standard procedure for screening thyroid dysfunction in preterm infants, and it was recommended to perform a second newborn screens (NBS) in infants born with GA < 32 weeks at 2 to 4 weeks after birth (Rose et al., 2023). Thyroid function parameters, including TT3, TT4, FT3, FT4, and TSH, were measured on a Roche Cobase 602 analyzer using the electrochemiluminescence immunoassay (Zhang et al., 2023).

### Neurodevelopment assessment

Neurodevelopment was predicted by analyzing general movements (GMs), which were scored from video recordings according to Prechtl’s General Movements Assessment (GMA). GMs was performed in the BPD group and the non-BPD group at the PMA of 36 weeks and 40 weeks. GMs were categorized as normal or abnormal and scored by assessors with advanced GMA certification who were unaware of the infant’s clinical history (Olsen et al., 2016).

### Statistical analysis

The study cohort’s characteristics were presented as median, means, standard deviation, frequency, or percentage. SPSS version 21.0 software was utilized for data analysis. Continuous variables with a normal distribution were assessed using the t-test, one-way ANOVA test, or repeated-measures ANOVA test, and the Mann-Whitney U test was used for continuous variables without a normal distribution. Categorical variables were analyzed using the chi-square or Fisher’s exact test. Statistical significance was defined as a *P*-value < 0.05.

## Results

### Clinical characteristics of the preterm infants

A total of 125 extremely and very preterm infants born at 26–32 weeks of GA were enrolled in the study, among which 40 (32%) infants developed BPD. The clinical features of the preterm neonates are presented in **Table 1**. The mean GA and mean birth weight of the BPD group were significantly lower than those of the non-BPD group (*P* < 0.0001). No significant differences were observed between the two groups in terms of gender, mode of delivery, antenatal steroid usage, maternal gestational diabetes mellitus, gestational hypertension, maternal thyroid disease, small for gestational age, or additional recorded newborn disorders, including sepsis, necrotizing enterocolitis, and retinopathy of prematurity (*P* > 0.05). The percentage of infants in the BPD group who needed caffeine and surfactant, as well as those who needed mechanical ventilation for longer than 14 days, was significantly higher than that of the non-BPD group (*P* < 0.05). Morbidities sush as HSPDA, and intraventricular hemorrhage were more frequent in the BPD group than in the non-BPD group (*P* < 0.05). Among the 40 BPD infants, 27 and 13 infants were classified as grade 1 and grade 2 BPD, respectively; none of the infants were classified as grade 3 BPD.

**Table 1:** Clinical features of the BPD and non-BPD groups.

Variables	Non-BPD (n = 85)	BPD (n = 40)	*P*-value
Gestational age, weeks	29.7 ± 1.7	28.9 ± 1.3	0.0061
Birth weight, g	1,385.0 ± 300.2	1,167.0 ± 171.6	< 0.001
Male, n (%)	41(48.2%)	21 (52.5%)	0.656
Cesarean section, n (%)	48 (56.5%)	25(62.5%)	0.523
Multiple pregnancy, n (%)	14 (16.5%)	6 (15.0%)	0.834
Antenatal steroid, n (%)	72 (85.5%)	35 (87.5%)	0.678
GDM, n (%)	15 (17.6%)	5 (12.5%)	0.891
Gestational hypertension, n (%)	9 (10.6%)	5(12.5%)	0.752
Maternal thyroid disease, n (%)	6 (7.1%)	2 (5.0%)	0.661
SGA, n (%)	6(7.1%)	7 (17.5%)	0.074
Caffeine use, n (%)	49 (57.6%)	31 (77.5%)	0.031
Surfactant use, n (%)	43 (50.6%)	30 (80.0%)	0.010
Mechanical ventilation > 14 days, n (%)	13 (15.3%)	14(35.0%)	0.012
Sepsis, n (%)	5 (5.9%)	6 (15.0%)	0.093
Hemodynamically significant PDA, n (%)	22 (25.9%)	21 (52.5%)	0.003
NEC, n (%)	2 (2.4%)	3 (7.5%)	0.170
Intraventricular hemorrhage, n (%)	22 (25.9%)	19 (47.5%)	0.016
Retinopathy of prematurity, n (%)	2 (2.4%)	4 (10.0%)	0.062

GDM, gestational diabetes mellitus; SGA, small for gestational age; PDA, patent ductus arteriosus; NEC, Necrotizing enterocolitis.

### Time-based distribution pattern of THs in the BPD and non-BPD infants

**Figure 2:**
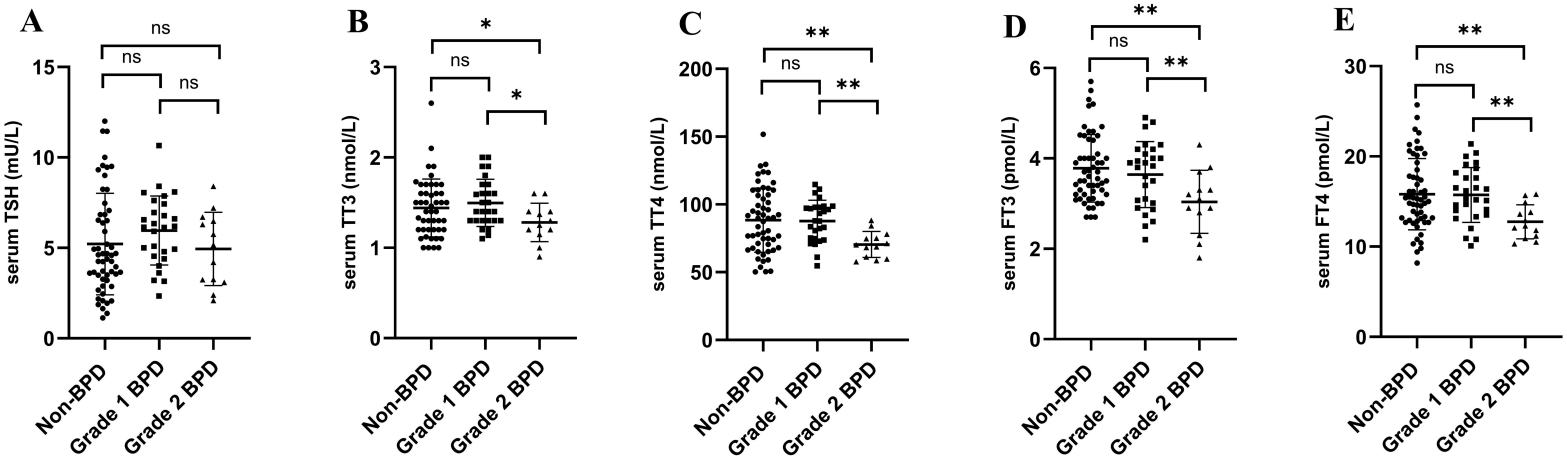
Serum thyroid hormone levels of the BPD and the non-BPD groups at 2 weeks after birth. **(A)** Serum levels of thyroid stimulating hormone (TSH) in the three groups. **(B)** Serum levels of total thyroxine (TT3) in the three groups. **(C)** Serum levels of total triiodothyronine (TT4) in the three groups. **(D)** Serum levels of free thyroxine (FT3) in the three groups. **(E)** Serum levels of free triiodothyronine (FT4) in the three groups. *P < 0.05; **P < 0.01; ***P < 0.001.

The distribution pattern of thyroid parameters was compared between the grade 1 and the grade 2 BPD groups (**Table 2**). At 2 weeks after birth, preterm infants born at <32 weeks of GA underwent screening for thyroid dysfunction following the standard NICU protocol. Serum TT3, TT4, FT3 and FT4 values were significantly lower in the grade 2 BPD group than in the grade 1 BPD group (*P* < 0.05; **Figure 2**). However, no statistical difference was found between the grade 1 BPD group and the non-BPD infants in terms of thyroid parameters (*P* > 0.05; **Figure 2**).

**Table 2:** Clinical features of the BPD groups.

Variables	Grade 1 BPD (n = 27)	Grade 2 BPD (n = 13)	*P*-value
Gestational age, weeks	29.0 ± 1.4	28.7 ± 1.3	0.491
Birth weight, g	1,207.0 ± 170.4	1,116.0 ± 168.0	0.123
Male, n (%)	13 (48.1%)	8 (61.5%)	0.427
Cesarean section, n (%)	16 (59.3%)	9 (69.2%)	0.542
Multiple pregnancy, n (%)	4 (14.8%)	2 (15.4%)	0.962
Antenatal steroid, n (%)	25 (92.6%)	15 (76.9%)	0.160
GDM, n (%)	4 (14.8%)	1 (7.7%)	0.523
Gestational hypertension, n (%)	3 (11.1%)	2 (15.4%)	0.702
Maternal thyroid disease, n (%)	1 (3.7%)	1 (7.7%)	0.588
SGA, n (%)	3 (11.1%)	4 (30.8%)	0.125
Caffeine use, n (%)	20 (74.1%)	11 (84.6%)	0.920
Surfactant use, n (%)	19 (70.4%)	11 (84.6%)	0.330
Mechanical ventilation > 14 days, n (%)	8 (29.6%)	6 (46.2%)	0.305
Sepsis, n (%)	3 (11.1%)	3 (23.1%)	0.321
Hemodynamically significant PDA, n (%)	12 (44.4%)	9 (69.2%)	0.141
NEC, n (%)	1 (3.7%)	2 (15.4%)	0.189
Intraventricular hemorrhage, n (%)19	10 (37.0%)	9 (69.2%)	0.056
Retinopathy of prematurity, n (%)4	2 (7.4%)	2 (15.4%)	0.408

**Figure 3:**
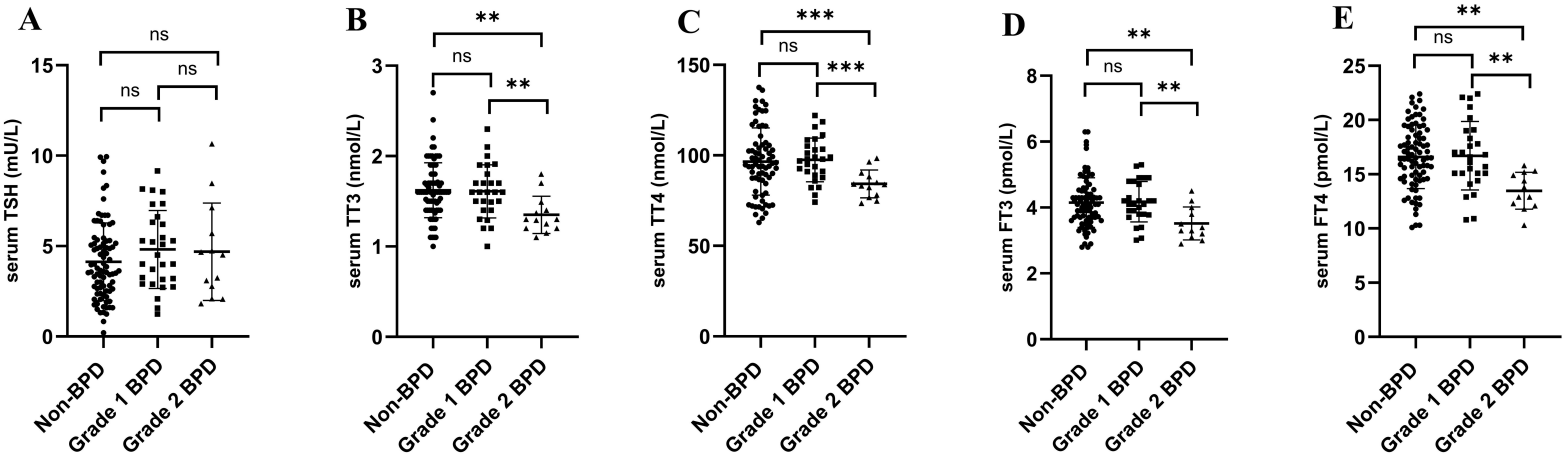
Serum thyroid hormone levels of the BPD and non-BPD groups at 4 weeks after birth. **(A)** Serum levels of thyroid stimulating hormone (TSH) in the three groups. **(B)** Serum levels of total thyroxine (TT3) in the three groups. **(C)** Serum levels of total triiodothyronine (TT4) in the three groups. **(D)** Serum levels of free thyroxine (FT3) in the three groups. **(E)** Serum levels of free triiodothyronine (FT4) in the three groups. *P < 0.05; **P < 0.01; ***P < 0.001.

As THs may vary with GA, we further evaluated TH values at 4 weeks after birth in the BPD and non-BPD groups. TT3, TT4, FT3 and FT4 levels remained significantly lower in the grade 2 BPD group than in the grade 1 BPD group (*P* < 0.05) at 4 weeks after birth, indicating inadequate TH secretion in the grade 2 BPD group (**Figure 3**). Notably, the grade 1 BPD and non-BPD groups exhibited comparable TH levels (*P* > 0.05; **Figure 3**).

### Physical and neurological developments of the BPD and non-BPD premature infants

The short-term physical and neurological developments of premature infants were shown in **Table 3**. At 36 weeks of PMA, the three groups exhibited no significant differences in their body weight (*P* = 0.443). Consistently, the incidence of extrauterine growth retardation was comparable among the three groups (*P* = 0.732). GMA was used to predict neurological development in early infancy. Compared with the grade 2 BPD group, the non-BPD and grade 1 BPD groups displayed a trend of better neurological development at 36 weeks and 40 weeks of PMA, although the difference was not significant (*P >* 0.05).

**Table 3:** Physical and neurological developments of premature neonates.

Variables	Non-BPD (n = 85)	Grade 1 BPD (n = 27)	Grade 2 BPD (n = 13)	*P*-value
Weight at 36 weeks’ PMA, g	2,027.7 ± 256.3	2,018.9 ± 372.4	1,916.1 ± 339.9	0.443
EUGR at 36 weeks’ PMA, n (%)	32 (37.6%)	12 (44.4%)	6 (46.1%)	0.732
Abnormal GMs at 36 weeks’ PMA, n (%)	61 (71.8%)	20 (74.1%)	13 (100%)	0.059
Abnormal GMs at 40 weeks’ PMA, n (%)	46 (54.1%)	15 (55.6%)	11 (84.6%)	0.113

PMA, Postmenstrual age; EUGR, Extrauterine growth retardation; GMs, General Movements.

## Discussion

Premature infants are particularly vulnerable to thyroid abnormalities (Klosinska et al., 2022). This study is among the first to show a graded relationship between the thyroid hormone deficiency and the BPD development in very preterm and extremely preterm infants at two different postnatal intervals (Vamesu et al., 2023). Particularly, we investigated the distribution pattern of TH levels in grade 1 and grade 2 BPD infants. The results revealed that preterm neonates with grade 2 BPD have underdeveloped hypothalamic-pituitary-thyroid axis, as demonstrated by lower TT3, FT3, TT4, and FT4 levels; this observation aligns with previous studies that identified THs as critical regulators of lung development and function (Dilli et al., 2010; Korkmaz et al., 2018). THs are essential for regulating mitochondrial pathways and biogenesis (Yu et al., 2018). Premature infants are more prone to diseases triggered by oxidative stress due to their underdeveloped antioxidant defense system, which may be partially caused by thyroid function deficiency (Wang and Dong, 2018). The present study reveals a significant association between thyroid dysfunction and the severity of BPD in premature infants, which was consistent with the previous study that showed that transient hypothyroxinemia of prematurity was associated with early lung morbidities, including neonatal respiratory distress syndrome and moderate to severe BPD (Dursun and Ozcabi, 2021). Additionally, various risk factors, including lower GA, respiratory distress syndrome, mechanical ventilation, and patent ductus arteriosus are associated with transient hypothyroxinemia of prematurity (Carrascosa et al., 2008), which aligns with the present study, wherein the BPD group had higher rates of surfactant use, prolonged mechanical ventilation, HSPDA, and intraventricular hemorrhage compared with the non-BPD group, which might further impact TH levels.

In premature infants, serum THs tend to change dynamically with postnatal age (Dilli et al., 2010). It was recommended as a standard procedure to perform a second NBS for congenital hypothyroidism in infants born with GA < 32 weeks at 2 to 4 weeks after birth (Rose et al., 2023), therefore, we analyzed the TH levels of the preterm infants at 2 and 4 weeks after birth. Future prospective studies could investigate the potential utility and predictive value of earlier assessments, such as at 1 week after life, to further optimize clinical management strategies. At 4 weeks post-birth, the grade 2 BPD group still exhibited significantly lower T4 and T3 levels compared to the non-BPD and grade 1 BPD groups. A previous survey also confirmed the importance of the TT4 level in predicting BPD severity in extremely low birth weight infants (Vamesu et al., 2023). Current guidelines recommend TH replacement therapy only for preterm infants diagnosed with congenital hypothyroidism (Klosinska et al., 2022). However, whether and when to start TH replacement therapy for such cases remains controversial. It was known that poor neurodevelopment more commonly occurred in BPD infants (Martin et al., 2023). The first two weeks after birth was a critical period for brain neurodevelopment and synaptogenesis (Klosinska et al., 2022). According to our results, the neurodevelopmental deficits observed at 36 and 40 weeks of PMA might be attributed to the relatively insufficient secretion of THs in the grade 2 BPD group. However, this hypothesis necessitates validation through long-term follow-up studies.

Although the present study is the first to comprehensively evaluate the role of THs in BPD development, it has some limitations. Specifically, the small sample size and retrospective design preclude us from establishing a definitive TH level cutoff value for implication in BPD onset. Despite this limitation, our findings revealed that insufficient TH levels in the early stage of life may be associated with more severe BPD and highlighted the importance of monitoring TH levels to potentially mitigate the risk of delayed neurodevelopmental outcomes in premature infants born at < 32 weeks of GA. Even though the trend toward poorer neurodevelopment in the grade 2 BPD group did not reach statistical significance, likely due to the small sample size in this subgroup, this calls for more research using a larger sample size and longer follow-up. Given the prominent impact of BPD on preterm infants, further studies are needed to elucidate the underlying mechanisms of BPD development to design appropriate targeted interventions.

## Data Availability

The raw data supporting the conclusions of this article will be made available by the authors, without undue reservation.
